# Protein language models can capture protein quaternary state

**DOI:** 10.1186/s12859-023-05549-w

**Published:** 2023-11-14

**Authors:** Orly Avraham, Tomer Tsaban, Ziv Ben-Aharon, Linoy Tsaban, Ora Schueler-Furman

**Affiliations:** 1 Department of Microbiology and Molecular Genetics, Faculty of Medicine, Institute for Biomedical Research Israel-Canada, The Hebrew University of Jerusalem, Jerusalem, Israel; 2 Gaffin Center for Neuro-Oncology, Sharett Institute for Oncology, Hadassah Medical Center and Faculty of Medicine, Hebrew University of Jerusalem, Jerusalem, Israel; 3 The Wohl Institute for Translational Medicine, Hadassah Medical Center and Faculty of Medicine, Hebrew University of Jerusalem, Jerusalem, Israel

**Keywords:** Protein language models, Natural language processing, Protein quaternary state, Oligomeric state prediction, Multilayer perceptron, Deep learning

## Abstract

**Background:**

Determining a protein’s quaternary state, *i.e.* the number of monomers in a functional unit, is a critical step in protein characterization. Many proteins form multimers for their activity, and over 50% are estimated to naturally form homomultimers. Experimental quaternary state determination can be challenging and require extensive work. To complement these efforts, a number of computational tools have been developed for quaternary state prediction, often utilizing experimentally validated structural information. Recently, dramatic advances have been made in the field of deep learning for predicting protein structure and other characteristics. Protein language models, such as ESM-2, that apply computational natural-language models to proteins successfully capture secondary structure, protein cell localization and other characteristics, from a single sequence. Here we hypothesize that information about the protein quaternary state may be contained within protein sequences as well, allowing us to benefit from these novel approaches in the context of quaternary state prediction.

**Results:**

We generated ESM-2 embeddings for a large dataset of proteins with quaternary state labels from the curated QSbio dataset. We trained a model for quaternary state classification and assessed it on a non-overlapping set of distinct folds (ECOD family level). Our model, named QUEEN (QUaternary state prediction using dEEp learNing), performs worse than approaches that include information from solved crystal structures. However, it successfully learned to distinguish multimers from monomers, and predicts the specific quaternary state with moderate success, better than simple sequence similarity-based annotation transfer. Our results demonstrate that complex, quaternary state related information is included in such embeddings.

**Conclusions:**

QUEEN is the first to investigate the power of embeddings for the prediction of the quaternary state of proteins. As such, it lays out strengths as well as limitations of a sequence-based protein language model approach, compared to structure-based approaches. Since it does not require any structural information and is fast, we anticipate that it will be of wide use both for in-depth investigation of specific systems, as well as for studies of large sets of protein sequences. A simple colab implementation is available at: https://colab.research.google.com/github/Furman-Lab/QUEEN/blob/main/QUEEN_prediction_notebook.ipynb.

**Supplementary Information:**

The online version contains supplementary material available at 10.1186/s12859-023-05549-w.

## Background

Proteins are the “working class” of living cells, performing many of the functions critical for life. Learning molecular details about the structure and function of a protein is often a difficult task, as this entails low-throughput and rigorous work. The quaternary state (qs), *i.e.*, the number of units assembling together to form a functional unit is an important characteristic of a protein. Many proteins form oligomers to carry out their molecular assignments [1]. These oligomers can be of homo- or hetero-oligomeric nature, *i.e.*, identical subunits or different subunits, respectively. The oligomeric formation can be obligatory or dynamic [2], and is often crucial for the proper activity of the complex. Examples for oligomeric proteins include the homotetrameric β-galactosidase [3], and the homotrimeric HTRA1 protease [4], where for both the homo-multimer formation is essential for their activity.

**Fig. 1:**
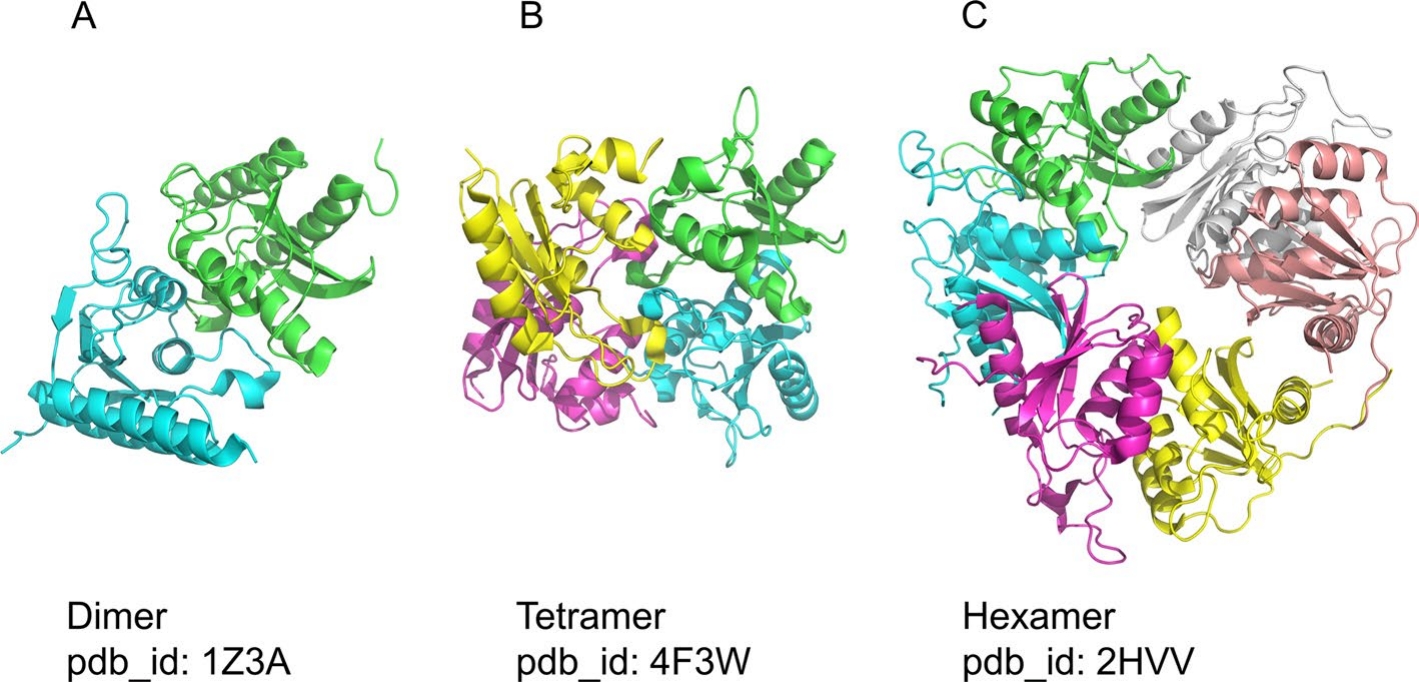
Proteins from the same fold family can assume different quaternary states (qs) All three proteins belong to the “Cytidine and deoxycytidylate deaminase zinc-binding region” family (ECOD *dCMP_cyt_deam_1*, f_id: 2492.1.1.5), but form either a dimer **A**, tetramer **B** or hexamer **C**. This demonstrates the complexity of determining qs, as members of the same structural family may form different states.

Proteins can be classified into families, whether functional (such as GO [5, 6] or KEGG [7]), structural (such as ECOD [8], PFAM [9], CATH [10]) or other classifications (e.g. sequence similarity). These classifications are important when comparing proteins to similar members of the relevant cluster. In this context, families of proteins may all adopt the same qs, or alternatively, may contain members that form various qs (Fig. 1). This adds a tier of complexity to qs determination, as simply learning or annotation transfer within families will not always yield the correct assignment.

Usually, experimental approaches are used to determine the qs, such as SEC-MALS, IEX-MALS, ultracentrifugation, to name a few. To alleviate these often expensive and laborious approaches, computational protocols have also been developed for this task, mostly starting from a solved crystal structure [11]. As an example, the widely used PISA protocol determines the most likely biologically relevant assembly based on the calculated strength of all different contacts among monomers in a solved structure [12]. However, PISA has several drawbacks, the main one being its dependency on information from a solved multimeric structure, where it chooses the correct assembly from within the oligomers in the crystal lattice. Another experiment-based qs predictor, EPPIC takes into account evolutionary information to ascertain the correct qs from a crystal lattice or other solved structures [13]. Another tool, GalaxyHomomer, also utilizes solved structures (or generates a model, if no solved structure is available), by selecting the best option among complexes generated by template based docking and *ab-initio* modeling [14]. The PROTCID database contains annotations about the quaternary structure of a protein monomer that is based on recurring monomer interactions in distinct solved structures of a protein [15]. Finally, QSbio takes into account information about qs of homologs retrieved from several data repositories [16]. This is done by superposing complete complexes and assessing the correct qs by exploiting the available evolutionary information. As for many other structural tasks, AlphaFold has been used in this context as well, where the confidence measures pTM, pLDDT and PAE are used to assess the most probable multimeric state [2, 17, 18], or by generating and scoring a variety of complex structures [19].

While it is quite natural to use structural information to elucidate the qs of a protein, there are also disadvantages to the above approaches, in particular, the computationally heavy structure prediction when no experimentally solved structure is available. How far can we then get using only sequence information? A straight-forward way would be to infer qs based on the qs reported for the protein family to which the protein belongs, or from the closest homolog, when available. However, a given protein family may host structurally similar proteins with distinct qs (as, e.g., in Fig. 1). How then should the qs be determined from sequence only? More sophisticated sequence representations could help address this challenge.

Learning information about a protein from its sequence is a well-established approach in protein annotation, as for example the use of multiple sequence alignments to extract evolutionary information [20]. Nonetheless, the construction of multiple sequence alignments is costly in time and computing power, and dependent on the availability of many homologous sequences, and is thus inherently biased towards evolutionary conservation. To address these problems and others, Natural Language Processing concepts have been applied to protein sequences, generating protein language models (pLMs) [21–23]. Briefly, a Neural Network is trained on a tremendous amount of protein sequences, learning the connection between the residues, and more specifically, their contextual meaning. Once learned, information can be extracted as embeddings (*i.e.* a numeric vector representation of the protein). These embeddings are subsequently used in a transfer learning step as input for supervised learning. The resulting embeddings are implemented for various tasks, ranging from predicting the protein secondary structure, localization and characteristics, to predicting three dimensional models [23–25]. The embeddings are very powerful, which stems in part from the fact that training was carried out when generating the embeddings, therefore the transfer learning step can be performed on a (relatively) small dataset (e.g. [26]).

**Fig. 2:**
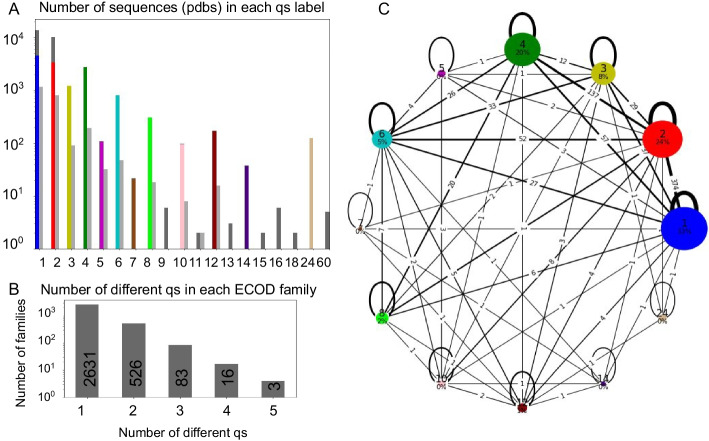
Overall data statistics and distribution of the quaternary state dataset used in this study **A**. Distribution of qs: For each qs (x-axis) the number of entries is shown (y-axis, in log-scale). Left and right bars show the training set (in color, after down-sampling; dark gray: samples removed for training) and hold-out set (light gray), respectively. Down-sampling was necessary due to the uneven distribution of the data, which is significantly skewed towards monomers, followed by dimers, trimers, tetramers and hexamers. **B**. Distribution of count of families (y-axis) with different qs within a given ECOD family (x-axis). While most families show the same qs for all their entries, a significant fraction contains a diverse set of qs (exact numbers are included in the boxes). **C**. Details of the composition of qs in different ECOD families, shown as a network, where the nodes represent different qs (colored as in A.), sized according to their amount (and percentage indicated). The edges represent families containing two different qs, with width proportional to the amount (and numbers indicated). Note that B and C show numbers in the training set, after down-sampling and removal of small groups.

Training a method for qs classification is challenging, for many reasons, in particular due to the inherently unbalanced data, with monomers outnumbering all other classes (in, e.g., the QSbio dataset; see, Fig. 2 below). The small classes have a dozen or even less entries, many of them with high sequence similarity, thus clustering together and providing less additional information. Moreover, the changes needed to shift a sequence from one qs to another may be very small, involving as few as 5 residues and in cases even a single point mutation [4, 27].

In this study we examine the power of pLM embeddings, derived from the pre-trained pLM ESM-2 [25], to capture and consequently classify the qs of proteins. We assemble our training set using curated data from QSbio, including only homomeric proteins, and using only high confidence entries. Our goal in this research is twofold: First, we wish to examine to what extent the embeddings hold the capacity to capture qs, if at all. For this purpose, we generate training and test sets, which are available for future research. Second, we built a useful classifier termed QUEEN (QUaternary state prediction using dEEp learNing), which could be employed both for low-throughput manual examination of protein sequences, and especially for high-throughput large scale classifications. One of the major advantages of using pLM is the speed of inference, once the embeddings have been generated. We show that ESM-2 embeddings can indeed be used for this task, with performance beyond that of simple sequence homology-based annotation transfer. We build a MultiLayer Perceptron (MLP) model using the embeddings as input, and explore the resulting classifier, its successful predictions and its failures.

## Results

*Organization of the dataset*: The QSbio database contains carefully curated information about the multimeric state of different proteins for which the structure has been determined [16]. The starting point of our study is a dataset extracted from QSbio, including only homomeric proteins of highest confidence, and only a single annotation per protein data bank (pdb [28]) entry (a total of 31,994 unique protein sequences, see Methods for full details). In this redundant dataset, each unique protein sequence is included as a separate entry, where very similar sequences will most often, but not always, have the same qs annotation. We separated this dataset into a training and a validation (hold-out) set, so that sequences with over 30% sequence identity would be in the same set. We then used the structural domain-based ECOD database [8] to cluster the sequences into domain families (at the family structural similarity level, “f_id”) for their further investigation. Overall, this dataset covers 19 different qs, ranging from monomers to 60mer homopolymers (Fig. 2A), and many ECOD families contain representatives of various qs (Fig. 2B and C, see also Additional file 1: Table SI, Additional file 2: Table SII, for detailed information about the dataset and results, and Additional file 3: Figure S1).

**Fig. 3:**
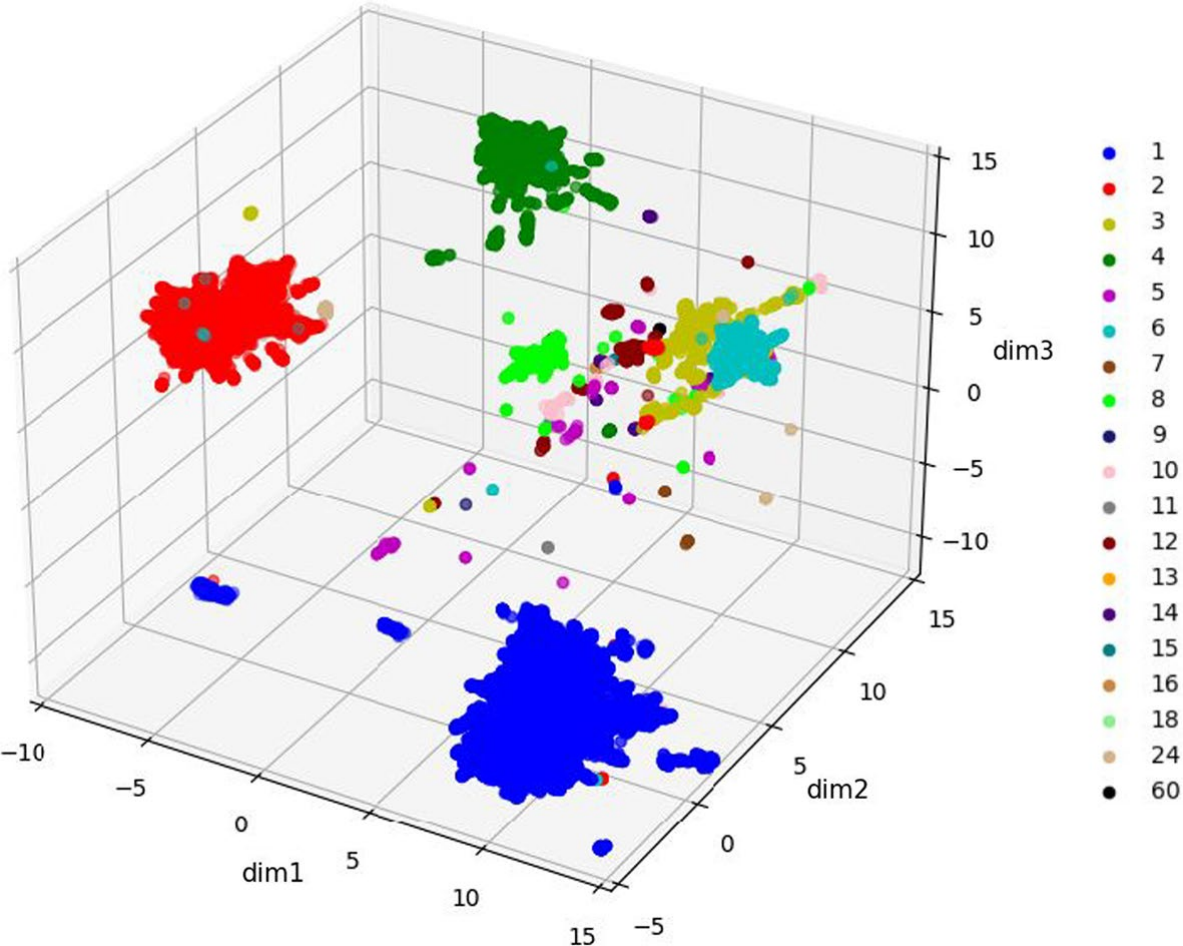
Capture of different qs by the pLM embeddings Supervised dimensionality reduction by UMAP is able to separate proteins of different qs, in particular for the qs represented by many entries in the dataset, with nice separation for octamers as well (light green). qs are colored as in Fig. 1A. Note that this map includes all the data, *i.e.,* both the training and hold out sets.

*Distinction of different qs by the language model:* First, we generated embeddings for each entry in the database. Each protein is represented by one embedding vector, which is obtained as the average of the vectors of the different residues in the protein sequence (see Methods for more details). In order to assess the capacity of pLMs to capture qs, we used supervised dimensionality reduction to visually demonstrate that the data indeed clusters by qs (Fig. 3). The large groups of labels (namely monomers, dimers, trimers, tetramers and hexamers), as well as some other qs are well separated on this map, demonstrating the model’s ability to characterize distinct features of each group. This suggests that the model could be used to predict the qs.

**Fig. 4:**
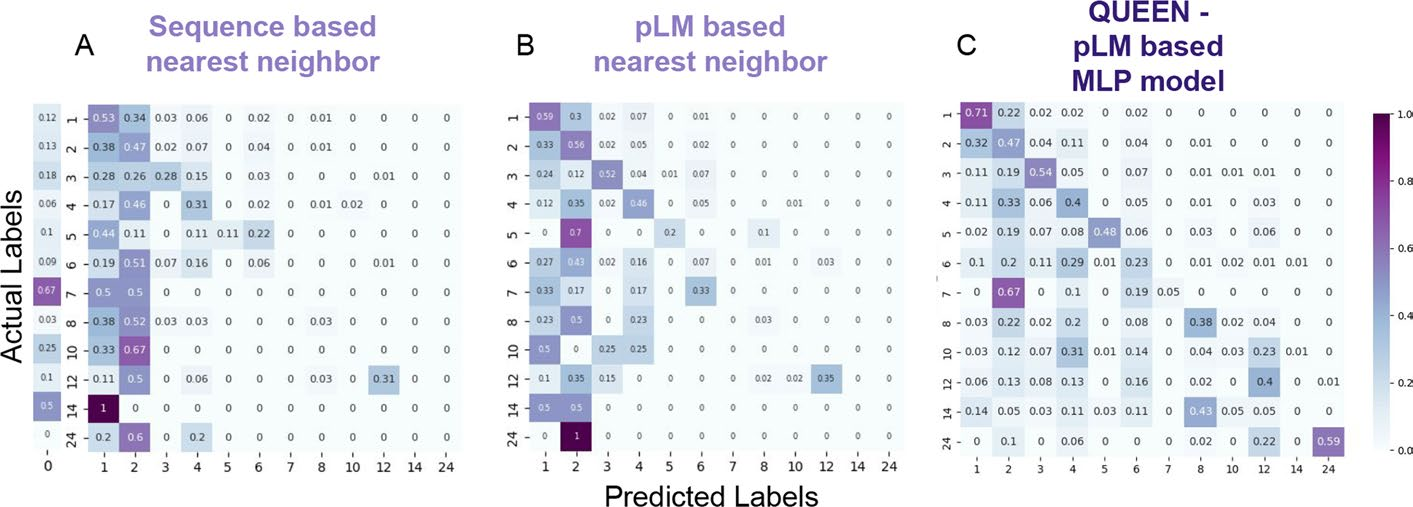
Accuracy of quaternary state prediction by different approaches The prediction is based on transfer of the qs annotation to each sequence in the test set based on **A**. the closest sequence in the train set (as determined by blast). The 0-predicted column indicates the fraction without any significant blast hit, and consequently no prediction; **B**. the highest similarity in embedding space in the train set (*i.e.,* cosine similarity between embedded vectors); and **C**. QUEEN—a deep learning model trained on the embeddings (see Text). The confusion matrix includes the frequency of cells representing predicted vs. actual labels (on x and y-axes, respectively), where a matrix occupying only the diagonal represents full success, while off-diagonal values represent wrong predictions. The balanced accuracy increases from left to right as indicated by the darker diagonal, highlighting improved prediction when moving from sequence, to language model representation, to QUEEN, the MLP model. Results are shown for the test set, based on information learned from an independent training set. For corresponding confusion matrices on a redundant set containing also information of sequence similar proteins, see Additional file 1: Figure S2.

*Inferring qs by annotation transfer from similar proteins:* To assess the ability of QUEEN to correctly predict the qs of a protein, we used annotation transfer to assign a qs to each sequence in the test set (Fig. 4A, B and Additional file 1: Figure S2). In this approach, the qs is inferred from the most similar protein with available annotation, *i.e.* the nearest neighbor in the embedding space (calculated as cosine similarity, see Methods), similarly to previous studies [29]. This can be compared to a corresponding annotation transfer based on sequence similarity. Using the embedding space has advantages: a distance can be calculated between any two vectors even if they are very different, since they represent a feature of a protein sequence. This is in contrast to sequence representation that is residue-based, and therefore necessitates sequence alignment, which can be challenging when comparing distant sequences.

Annotation transfer using embedding distance outperformed corresponding annotation transfer based on sequence identity in predicting qs (Table 1). This applies to prediction with available prior knowledge (*i.e.,* on a redundant set that contains sequence-similar proteins, Additional file 1: Figure S2). Importantly, this holds also when prior knowledge is *not* available (*i.e.,* for the test set that does not contain any entry with significant sequence identity to the training set): The balanced accuracy increases from 0.15 to 0.23 (Table 1, compare Figs. 4A, B).

**Table 1:** Performance of different models for the prediction of quaternary states (qs). Balanced Accuracy and F1 scores

		Annotation transfer, based on		pLM MLP model, based on	
		Sequence	pLM	ESM-2 embeddings (QUEEN)	Protbert embeddings
No information about sequence homologs available ^1^	BA^2^	0.15	0.23	0.36	0.19
	F1^3^	0.43	0.54	0.52	0.41
Full homology information available	BA	0.6	0.67	–	–
	F1	0.79	0.85		

^1^ no sequence with > 30% sequence available to transfer from; ^2^BA: balanced accuracy; ^3^ F1: F1 score; Precision and Recall values are provided in Additional file 3: Table S3, and Precision-Recall and ROC curves are provided in Additional file 3: Figure S3. See Methods for definitions

**Fig. 5:**
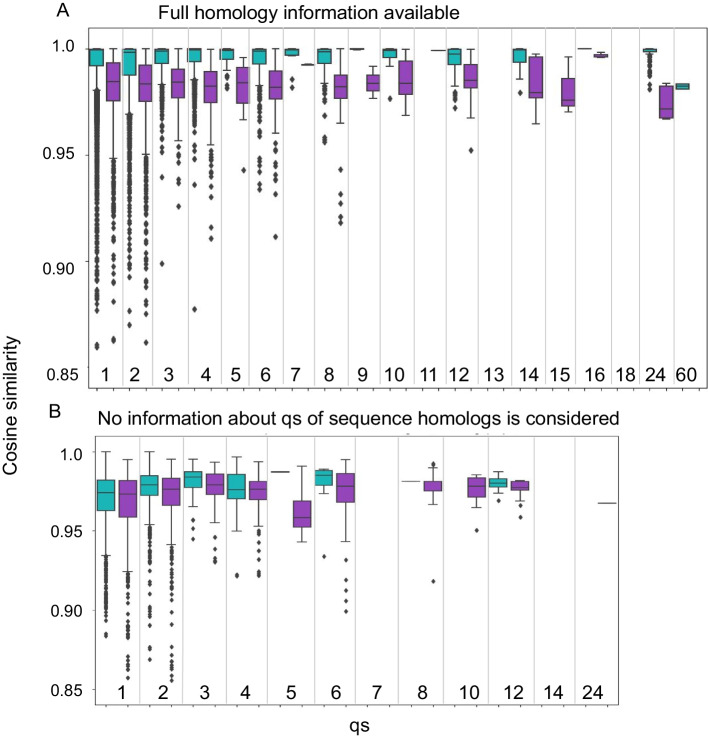
Cosine similarity provides an estimate of the reliability of qs predictions based on annotation transfer from homolog proteins **A**. Cosine similarity using information that includes qs of homologous sequences. For each qs (x-axis) a box plot depicts the distribution of the cosine similarities for correct (green) and incorrect (purple) qs predictions (y-axis). The plot is capped at 0.85, with 3 dots removed for clarity. The two distributions differ significantly (one sided Wilcoxon test, *p*-values < 0.05, except for qs = 7, *p*-value = 0.051; see Additional file 2: Table S1). This separation demonstrates the power of embeddings to capture qs, and can be used to assess the confidence of annotation transfers. **B**. Corresponding plot of cosine similarities when information of qs of homologous sequences is NOT included (*i.e.* no entry with > 30% sequence identity is considered for annotation transfer). In this case, the two distributions show no significant difference.

When the qs of a new sequence is predicted, it is often homologous to previously annotated sequences, which improves prediction (compare Additional file 1: Figure S2 and Fig. 4, and see Table 1). In such a setting, (*i.e.,* when including qs information of homologs), we observe a significant separation between cosine similarities used for transfer of correct and incorrect predictions (Fig. 5A; with the exception of qs = 7; Individual p-values are summarized in Additional file 1: Table S4). Thus, in these cases the cosine similarity can be used to assess whether simple annotation transfer may be sufficient to determine the qs. This is however not applicable for qs assignment without information from homolog proteins, as apparent in Fig. 5B.

*Training a model for qs prediction based on embeddings:* The above initial analysis suggests that the embeddings include information about qs. To optimally leverage their use, we trained an embedding-based MLP model to predict qs. We trained and performed hyperparameter tuning on 5 sets of cross-validation within our training set (each time training on 80% to predict a different 20% set, see Methods and Additional file 1: Figure S4) to define the final model and its parameters (Fig. 4C; Importantly, the hold-out set was not used at any step of training and validation of QUEEN). To achieve improved performance we experimented with several strategies, which highlighted the importance of down-sampling of monomers and dimers to obtain a more equilibrated dataset for training (resulting in similar size of monomer, dimer and tetramer qs, see Figs. 2A, C). Even after down-sampling, the resulting confusion matrix (Fig. 4C) still highlights the relatively high success rate of the monomer qs predictions. As for multimers, for many of the wrong predictions, dimers and tetramers are chosen, more so than monomers (see columns of dimer and tetramer predictions in Fig. 4C). This hints that QUEEN may have learned to detect multimerization rather than the exact qs, as shown in the striking example of the predominant classification of heptamers as dimers (see below).

Compared to nearest neighbor annotation transfer, QUEEN shows significant improvement (compare Fig. 4C to B; balanced accuracy 0.36 *vs.* 0.23, see Table 1). While success is not uniform across all labels, correct prediction (> 40% in the diagonal of Fig. 4C) is achieved in 7 out of the 12 trained and predicted qs classes. When examining the results with one representative per each ECOD family (*i.e.,* no redundant information) the balanced accuracy decreases to 0.26 ± 0.03. The difference in performance might be a result of the smaller size of the evaluated set.

**Fig. 6:**
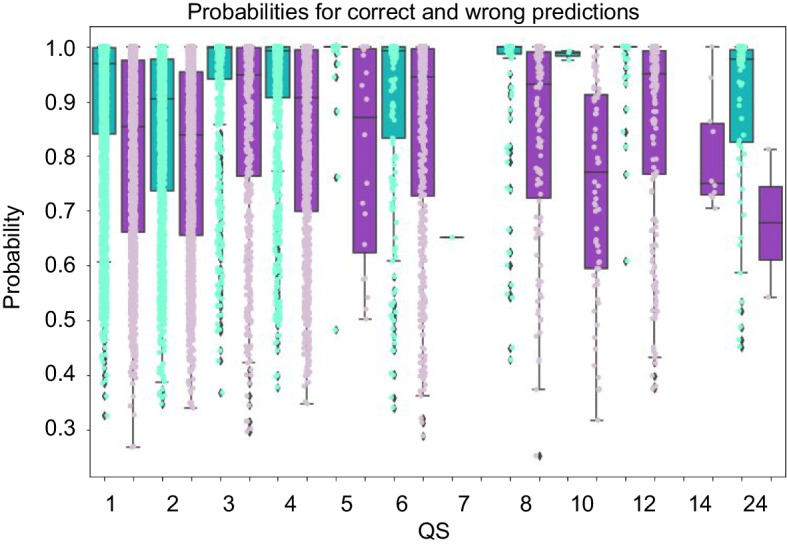
QUEEN predictions show consistently higher confidence for correct assignments For each qs (x-axis) box plots and dots show the distribution of the probabilities of correct (green) and incorrect (purple) predictions. Correct predictions are consistently predicted with higher probability than incorrect predictions, for all qs (one sided Wilcoxon test, *p*-value < 0.05). This separation can be used to assess correct predictions, also in the absence of homologs.

*Confidence of prediction as indication of success of QUEEN:* While QUEEN provides prediction of a specific qs, it is also interesting to examine the underlying probabilities that drive the label prediction and to assess whether they are indicative of successful *vs.* wrong predictions. Reassuringly, comparison of the distributions of the corresponding probabilities (*i.e.,* true-positives and false-positives) reveals a significant difference between the two (Fig. 6), with *p*-values ranging from 0.04 to 6*10^–88^ (Additional file 1: Table S5). Of note, in contrast to the pLM-based annotation transfer for which we can only provide a confidence estimate when information about the qs of homologous sequences is available (based on cosine similarity, Fig. 5), using the QUEEN MLP model, this is now also possible for qs predictions that are not based on homolog information (based on the predicted probability).

**Fig. 7:**
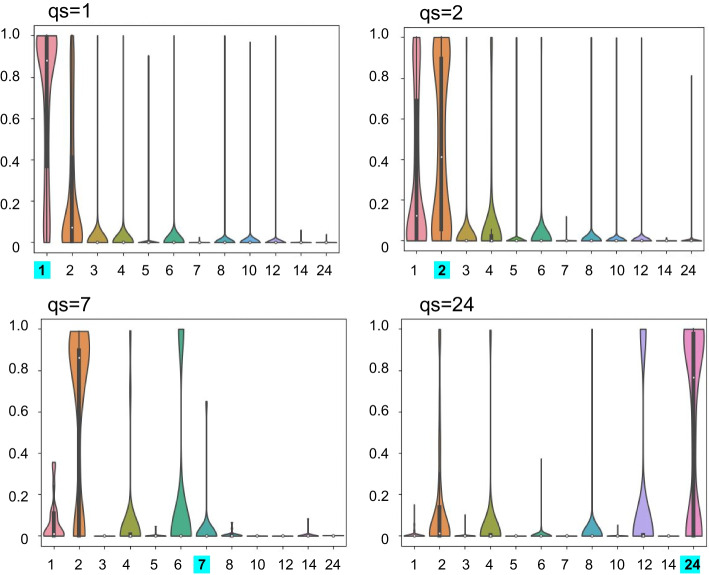
Distribution of predicted probability (‘confidence’) for representative qs categories The plots show violin plots of the prediction probabilities (y-axis) for different qs labels (x-axis), for actual qs = 1,2,7 and 24 (indicated in the title and highlighted in cyan on the x-axis).

The distributions of predicted qs shown in Fig. 7 reveal a clear preference for monomers for proteins that form monomers, as expected from the successful prediction for the qs of monomeric proteins (See Additional file 1: Figure S5 for full plots). In contrast, the distribution of qs predictions for heptamers (that QUEEN did not learn well, see Fig. 4C), is much more varied. However, as also apparent in the confusion matrix (Fig. 4C), the false negative heptamers are predicted to form different multimerization states, despite monomers being the largest class. This reinforces the notion that protein sequences hold information about multimerization, even though not always about the correct state. 24-mer is another interesting example, where besides high probability of 24-mers, the model also predicts the divisors of 24, with enrichment of the rare 8- and 12-mer classes.

**Fig. 8:**
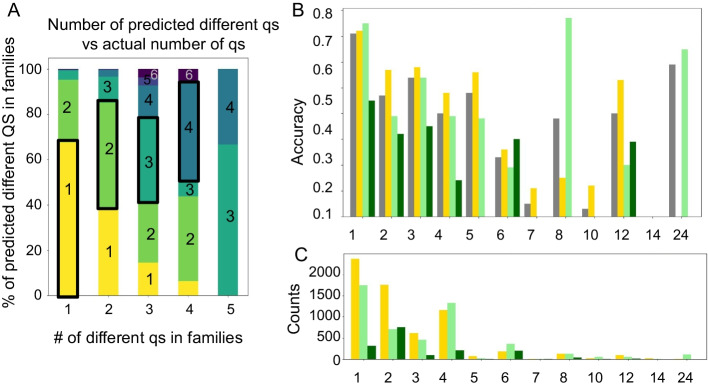
Prediction success and diversity of families with multiple qs **A**. QUEEN has an accurate representation of the degree of diversity of different families: Clusters with multiple qs are predicted as such. Shown are the percentage of families predicted to have different qs (y-axis) for a given number of distinct qs in a family (x-axis). Numbers of qs are indicated in the boxes and color-coded. **B**. Accuracy (y-axis) for different qs (x-axis). The different bars show four distinct groups within each qs: Overall accuracy (grey), families adopting a single qs (yellow), families adopting multiple qs—predominant qs (light green) and rare qs (dark green). **C**: Corresponding counts of the groups presented in B.

*Examination of diverse families*: Incorrect predictions shown in Fig. 7 could be a consequence of inaccurate learning for ECOD families that adopt more than one qs. We therefore compared performance for ECOD families for which all members adopt the same qs, to those that adopt diverse qs (families with single members were treated separately, as from one annotated sequence it cannot be concluded whether proteins in such a family can adopt different qs). Overall, the variation of qs within the family (*i.e.,* how many different qs it includes) is well captured by QUEEN (Fig. 8A). For homogenous families QUEEN predominantly predicts a single qs, while for families with more qs, the number of predicted different qs predominantly corresponds to the actual number of different qs. In this context, performance tends to be slightly improved for single qs families (Fig. 8B, C). Moreover, for families with diverse qs, predictions for proteins that adopt the dominant qs of that family are more accurate than corresponding predictions for proteins adopting outlier qs.

**Fig. 9:**
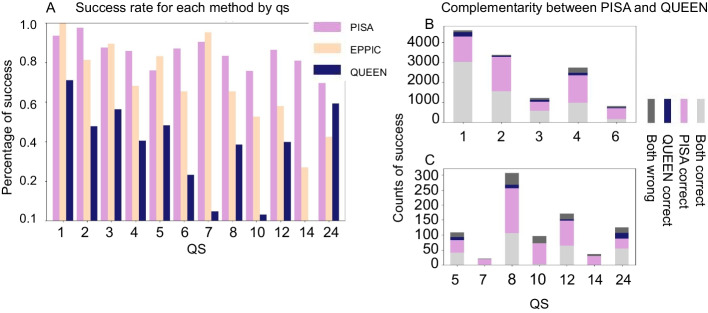
Comparison of success of qs predictions of different methods: Structure-based PISA, EPPIC, and sequence-based QUEEN. **A**. Success (y-axis) for different qs (x-axis) for the different approaches. PISA and EPPIC predominantly outperform QUEEN. Note that PISA and EPPIC use not only the structure but also the full crystal information, and they only choose from the available options within the crystal lattice. **B** and **C**. Success (y-axis) colored according to performance by PISA and QUEEN for different qs, highlighting the significant overlap, but also the additional potential contribution of QUEEN. The division between B and C is for clarity of the y-axis scale, B showing the larger classes 1–4, 6, and C showing the smaller classes 5, 7–24

*Comparison of QUEEN performance to other related approaches:* How well does this approach perform compared to other related methods? Comparison may provide new insight into the features that determine qs. Of note, to our knowledge, the present model is the first to use sequence information only, in contrast to other methods that all use a solved or predicted structure as input for qs determination or prediction. Not surprisingly therefore, PISA outperforms QUEEN for every class, and EPPIC shows a very similar trend (Note however the better performance of QUEEN for 24mers; Fig. 9A). Nevertheless, examination of the degree of complementarity of these approaches suggests that a significant fraction of proteins is correctly predicted only by QUEEN (5–20% for different qs; Fig. 9B and C). Therefore, a combination of the different approaches may be beneficial, pending our ability to identify the correct predictions.

*Comparison to other language models—Protbert:* We show here classification and qs assignment based on embeddings of the protein sequences. These embeddings were generated using the language model developed by Meta AI—ESM-2. We examined whether the source of the embeddings has an impact on the final results, and indeed it does. Repeating this protocol using embeddings from protbert_BFD resulted in worse performance, showing less improvement compared to simple sequence based nearest neighbor annotation transfer (see Table 1).

**Fig. 10:**
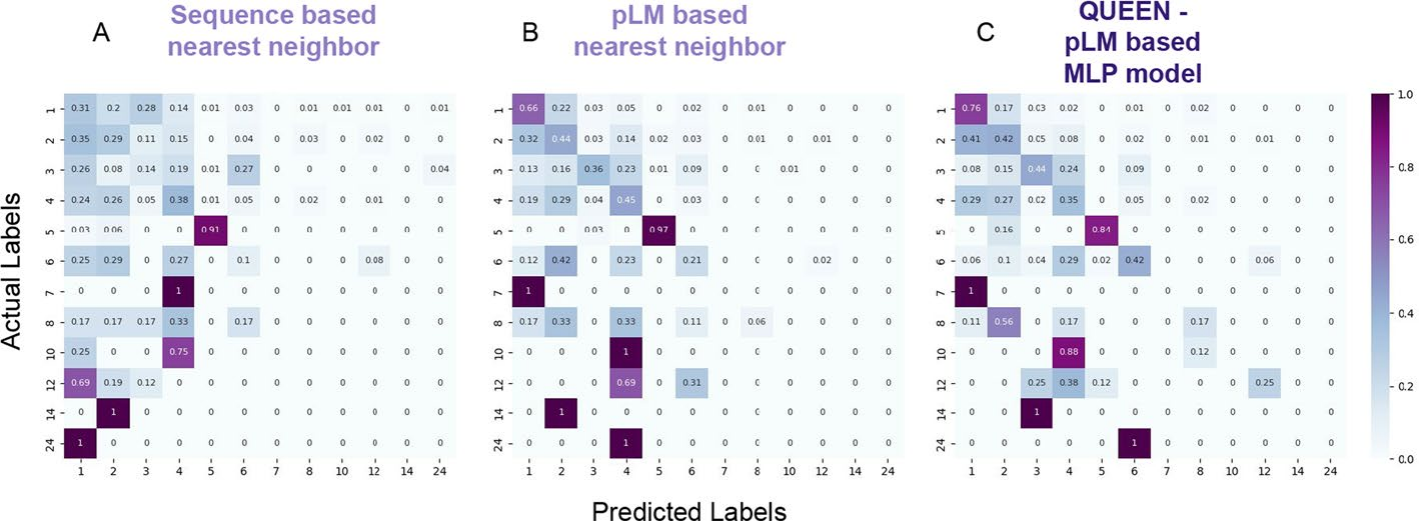
Accuracy of qs prediction by different approaches for the holdout set: The prediction is based on transfer of the qs annotation to each sequence in the holdout set based on **A**. the closest sequence in the training set (as determined by blast); **B**. The highest similarity in embedding space in the Training set (*i.e.,* cosine similarity between embedded vectors); and **C**. QUEEN trained on the embeddings (see Text). The confusion matrix includes the frequency of cells representing predicted *vs.* actual label (on x and y-axes, respectively), where a matrix occupying only the diagonal represents full success, while off-diagonal values represent wrong predictions. The balanced accuracy increases from left to right as indicated by the darker diagonal, highlighting improved prediction when moving from sequence, to language model representation, to the deep learning model

*Robustness of QUEEN performance demonstrated on the independent holdout set:* The results presented until now were calculated on the test sets of the 5 cross-validation runs. Based on these results, we used the optimized hyperparameter set to define our final model. To evaluate its performance, we now opened the hold out set that we had set aside at the beginning of our study. The performance using sequence or pLM-based annotation transfer on this (smaller) hold out set is slightly better (see Fig. 10 for confusion matrices and Table 2; compare to Fig. 3 and Table 1 above). Reassuringly, QUEEN performs similarly to what we report for the cross-validation performance, with balanced accuracy slightly lower at 0.3, and for most qs, correct predictions can be identified based on the estimated prediction probability, as observed during training (compare Additional file 1: Figure S6 to Fig. 6). A similar balanced accuracy is also obtained when examining one representative only (0.28 ± 0.05).

**Table 2:** Performance on the holdout set for the prediction of quaternary states (qs)

	Annotation transfer, based on		QUEEN
	Sequence	pLM	
BA	0.18	0.26	0.30
F1	0.34	0.55	0.58

## Discussion

Protein language models have proven to be most useful to the study of proteins, in particular due to the rich information that their embeddings can provide [30, 31]. In this work we explore the ability of protein embeddings to capture and classify quaternary states, qs. Our motivation is twofold: First, this allows us to understand how well information about the qs is represented in a sophisticated, yet sequence-based model that does not explicitly consider the protein structure as input. Second, we wished to provide a simple and fast tool to the public for the prediction of the qs of a protein based on its sequence only, to allow for its wide application and hopefully the acceleration of protein research.

Our analysis shows that QUEEN performs better than a sequence-based annotation transfer approach, as well as the use of pLM embeddings only, demonstrating that improved representation of sequence features, and additional learning can improve the representation of qs. Our analysis also demonstrates the difference in performance using different pLMs, as it is better than a corresponding Protbert-based pLM. Overall, QUEEN shows a good ability to distinguish between monomers and multimers. For seven qs success rate tops 40%, a performance which can likely be further improved. Future attempts to predict qs using pLM can profit from including structural information, such as features from a solved or predicted structure, or the use of structural embeddings. As an example, a recent study aimed at prediction of protein function has generated a model that learns PFAM domains, and uses these to infer functional annotation [32]. Given information on qs of a larger set of proteins, a similar approach could be implemented for qs prediction.

QUEEN has a “sense” of diversity, *i.e.,* sequences from the same family, despite having a similar sequence and similar fold, are not always classified with the same qs. In particular, the degree of variation of qs in different families is overall nicely captured by the model. Therefore, QUEEN could be useful to estimate the overall homogeneity of the qs within a new family. Nevertheless, as is also true for many other deep learning applications, QUEEN is not able to accurately predict qs changes that occur from point mutation (data not shown). Of note, QUEEN was trained on sequences extracted from solved structure rather than the full protein sequence as reported in uniprot [33] (for which the qs is not necessarily the same and known), and therefore it remains to be investigated how well it will perform for full sequences. In its current implementation, we suggest using it for the prediction of selected regions that mainly include defined domains, as QUEEN did not have the opportunity to train on unstructured regions that are not resolved in solved structures.

A number of qs prediction and assessment tools are already available. The widely used PISA strictly relies on a solved structure—not only the monomeric fold, but the entire complex. In this context, PISA calculates and determines the qs from within the available options in the complex. When a crystal structure is examined by PISA, only the combinations comprising the crystal lattice and the consequent complex are considered. This reduces, often dramatically, the qs that may be considered, improving predictions. Nonetheless, QUEEN succeeds in certain cases where PISA fails (Fig. 9 B and C). Pending our ability to reliably identify these cases, this could pave the way for further improvement by a pLM-based model such as QUEEN. Optimal extended leverage of structure prediction can be obtained by using state of the art structure prediction methods such as AlphaFold and ESMfold to provide structural information (e.g. [2]), in particular by modeling different qs to identify the most promising one [17].

Additional avenues remain to be explored, such as the investigation of more LMs for this task, besides ESM and ProtBert. Furthermore, a more complex model can be designed, by relating to the entire matrix rather than averaging the values. This work is merely the first step in using PLMs for qs prediction, demonstrating it is indeed possible.

## Conclusions

Protein sequences hold information regarding the protein’s quaternary structure. Leveraging this information can be directed towards predicting the qs from sequence alone, as shown here in detail. This work offers a useful tool for qs prediction, and opens the door to further investigation and improvement of utilizing pLMs to predict qs.

## Methods

### Aim, design and setting of study

The aim of this study is to develop a pLM sequence based MLP model for the prediction of the quaternary state of proteins (i.e., the number of monomers that assemble to generate a functional unit). We designed the study based on a filtered set of annotated proteins from the curated QSbio database: this set was divided into non-overlapping protein families to allow for the stringent evaluation of performance on an independent set of proteins. We compare this model to several other approaches on the same dataset, including annotation transfer based on (1) the most similar sequence with known qs, (2) the most similar pLM embeddings, and (3) a different pLM, Protbert_BFD; as well as predictions by established, structure-based protocols (4) PISA and (5) EPPIC.

### Dataset

The data used in this study is a subset of the data annotated in QSbio V6_2020, courtesy of Emmanuel Levy [16]. The data was filtered so that the remaining set met the following criteria: homomers (*i.e.,* comprised of copies of the same monomer), with qs annotation (that was not changed in an internal validation process, *i.e.,* “corrected_nsub” =  = “nsub”), and highest confidence level (best biological unit “Best_BU” =  = 1 and QSBIO_err_prob < 15). Duplicate fasta sequences were removed, retaining only the highest confidence annotation. This process resulted in a final set of 31,994 entries, spanning 19 qs labels. The sequences were taken from the solved PDB structure, including all residues present in the experimental construct. PISA and EPPIC performance were also extracted from this database.

### Generating independent train and hold-out sets

This filtered set above was split into a train and a hold-out set (using the *sklearn* function *model_selection.StratifiedGroupKFold* [34]. To prevent leakage between the data used for training and the used for final validation of the model (the hold-out set), we grouped the sequences by similarity (using MMseqs [35]), and clustered the data by 30% sequence identity with at least 30% coverage: *mmseqs easy-cluster* < *input_fasta.fasta* > *session* < *session_dir_location* > *–min-seq-id 0.3 -c 0.3 -s 8 –max-seqs 1000 –cluster-mode 1 –cluster-reassign.*

This relatively low coverage cut-off was selected to make sure the groups are indeed as separated as possible. Grouping of the data was done with the cluster representatives, defining the sets so that an entire cluster is included in the same set. We stratified the sets to ensure similar distributions of qs states in both sets, as much as possible.

About 10% of the data was put aside as a hold-out set. Within the remaining set we performed several cross-validation experiments for hyper-parameter tuning, model selection etc. For this, we divided this set into 5 sets of approximately similar size, and 5 cross-validation experiments were performed using each time a 80%-20% separation of training and test set (the approximation is due to the group and stratification constraints).

### Generation of embeddings and pooling

Embeddings from the ESM-2 model were generated locally, with no need for a GPU. The ESM-2 model generates a vector of length 1280 per each amino acid in each protein sequence, resulting in a matrix of size *L* × *1280* (L = sequence length). We performed mean pooling, averaging the matrix over the L dimension, to obtain a vector of 1280 for each sequence. This results in uniform embedding length for each entry.

Protbert_BFD embeddings were generated similarly, where the representation for each residue is a vector of length 1024, thus resulting in a vector representation of length 1024 per entry.

### Dimensionality reduction

We performed supervised dimensionality reduction with *UMAP* [36]. The parameters used are: *n_components* = *3, n_neighbors* = *350, min_dist* = *0.5.*

After obtaining the reduced vectors we plotted all three target dimensions for visualization.

### Nearest neighbor annotation

Comparison of the embeddings to the sequence was carried out by using the nearest neighbor algorithm to transfer the qs annotations. Annotation transfer was conducted in two parallel implementations: (1) without inclusion of homolog sequences for comparison and annotation transfer (*i.e.,* sequences in the test set were assigned a qs based on the closest entry in the train set). (2) including all close homolog sequences as well *(i.e.,* including sequences from the test set). The nearest neighbor was identified based on sequence similarity distance calculated with a local alignment as incorporated in MMseqs [35], with default parameters (sequence-based annotation transfer), or based on cosine similarity of the embedding vectors (for pLM-based annotation transfer).

### Hyperparameter tuning and MLP model

Exploring hyperparameters was done using *RandomizedSearchCV* from *sklearn* [34]. Among the hyperparameters used was the downsampling of the monomer and the dimer classes, which improved performance and was thus included in the pipeline. For downsampling we used the package *imblearn* and the function *RandomUnderSampler *[37]. The final downsampling factor chosen was 3, thus reducing to 33% the number of monomers and dimers in the training set.

The hyperparameter search was performed on the fivefold cross-validation described above, and the final parameters were selected based on the best results for adjusted balanced accuracy (see Additional file 1: Figure S4).

- Model parameters:

- MLPClassifier
- hidden_layer_sizes=(120,), learning_rate='adaptive', solver='adam',
- learning_rate_init=0.01, max_iter=1000, n_iter_no_change=20,
- random_state=22, tol=0.001)

- Sampled space (in bold: chosen parameter value):

- activation = [**'identity**', 'logistic', 'tanh', 'relu']
- learning_rate = ['constant', 'invscaling', **'adaptive'**]
- learning_rate_init = [0.001,0.005,** 0.01,** 0.05, 0.1]
- solver = ['sgd', **'adam'**]
- Max_iter = [100, 200, 300, 400, 500, 600, 700, 800, 900, **1000**]
- *n_iter_no_change* = [5, 10, 15, 20, 30, 40]
- tol = [0.0001, 0.0005, **0.001,** 0.01, 0.1, 1]
- hidden_layer_sizes = [(10,), (20,), (40,), (60,), (80,), (100,), **(120,)**, (140,), (160,), (180,), (200,), alpha = [0.0001, **0.0005,** 0.001, 0.01, 0.1, 1]
- batch_size = [10, 50, 100, 150, 200, **250,** 400, 600, 1000, 1500, 2000, 5000]

## Statistical analysis

The statistical analysis was carried out using a one-sided (“greater”) Wilcoxon test, implemented through *scipy.stats.ranksums*, with *alternative* = *'greater'*, the correct predictions passed as x and the wrong predictions passed as y.

### Packages and versions

All the scripts were written using *Python3.7*.

Embedding the sequences was carried out using *Torch*. The ESM-2 model used is EsmForSequenceClassification, with the specific version of *esm2_t33_650M_UR50D()* [25]. We initialized the parameter called "problem_type" to be "multi label”.

Embedding with Protbert was done using BertModel and the specific version *prot_bert_bfd* [23].

*MMseqs* version ad5837b3444728411e6c90f8c6ba9370f665c443 was used, installed locally.

*UMAP* version 0.5 was used.

For analyses and data handling we used *pandas* version 1.3.5 [38].

Graphs and plots were generated with *matplotlib* and *seaborn*, versions 3.5.3 and 0.12.0 [39, 40].

The network plot was generated using *networkx* version 3.0.

### Pymol

Protein structure visualization was done using *PyMol* version 2.2.0 Open-Source.

### Supplementary Information

**Additional file 1**: Supplementary Table SI:  Embeddings.

**Additional file 2**: Supplementary Table SII:  Model Results. 

**Additional file 3**: Supplementary Material. Contains Supplementary Figures S1-S6 and Supplementary Tables SIII-SV.

## Data Availability

All data is provided as supplementary tables in csv format. The data is also available in the github repository: https://github.com/Furman-Lab/QUEEN.

## Abbreviations

Qs: Quaternary state
pLM: Protein language model
QUEEN: QUaternary state prediction using dEEp learNing
MLP: MultiLayer Perceptron
BA: Balanced accuracy
