# Supplementary material for: Protein language models can capture protein quaternary state
Source: BMC Bioinformatics. 2023 Nov 14;24:433. doi: 10.1186/s12859-023-05549-w (PMC10647083; doi:10.1186/s12859-023-05549-w)
Supplement: Supplementary file 3 — Additional file 3: Supplementary Material. Contains Supplementary Figures S1-S6 and Supplementary Tables SIII-SV. [file 12859_2023_5549_MOESM3_ESM.pdf]

Supplementary Material

Supplementary Figures

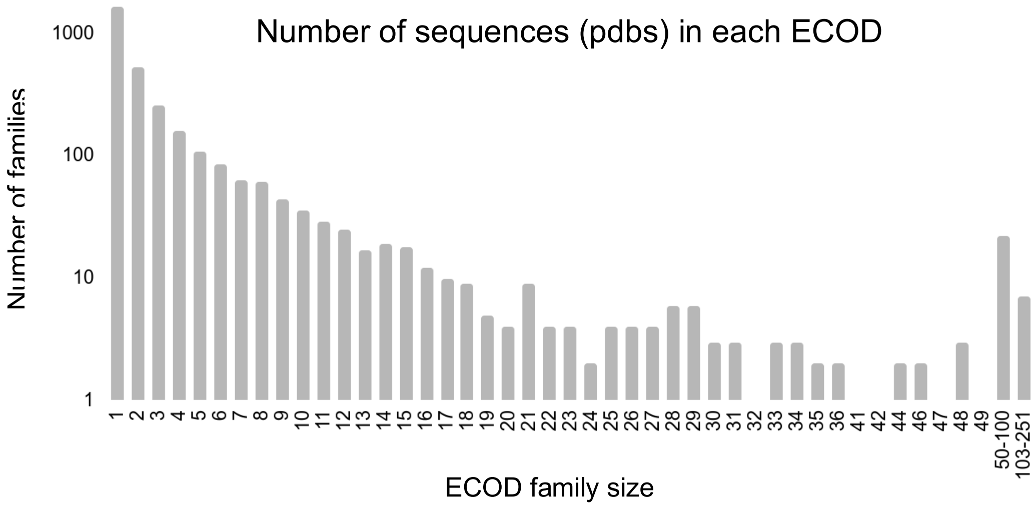

Supplementary Figure S1: ECOD family sizes. X-axis shows the family size, y-axis the number of families of the corresponding size. Large families are grouped together for clarity. Accompanies Figure 2.

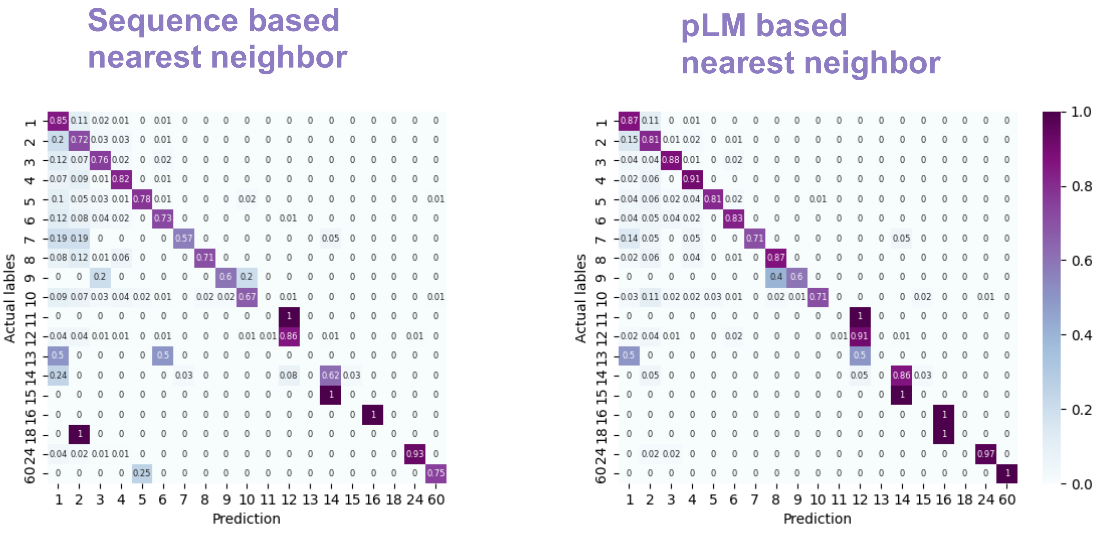

Supplementary Figure S2: Success of nearest neighbor annotation transfer when close homologs are available. **A.** Sequence-based annotation transfer (balanced accuracy, BA=0.6), **B.** Cosine similarity-based annotation transfer (BA=0.67). Accompanies Figures 4A and B, respectively.

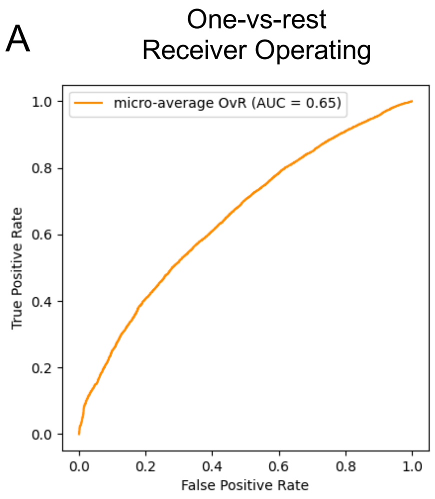

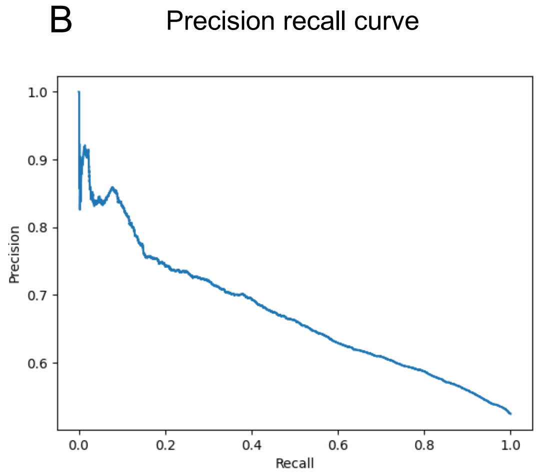

Supplementary Figure S3: Model evaluation parameters.
A. Receiver Operating Characteristic curve, calculated by the one-vs-rest (OvR) method.
B. Precision recall curve.

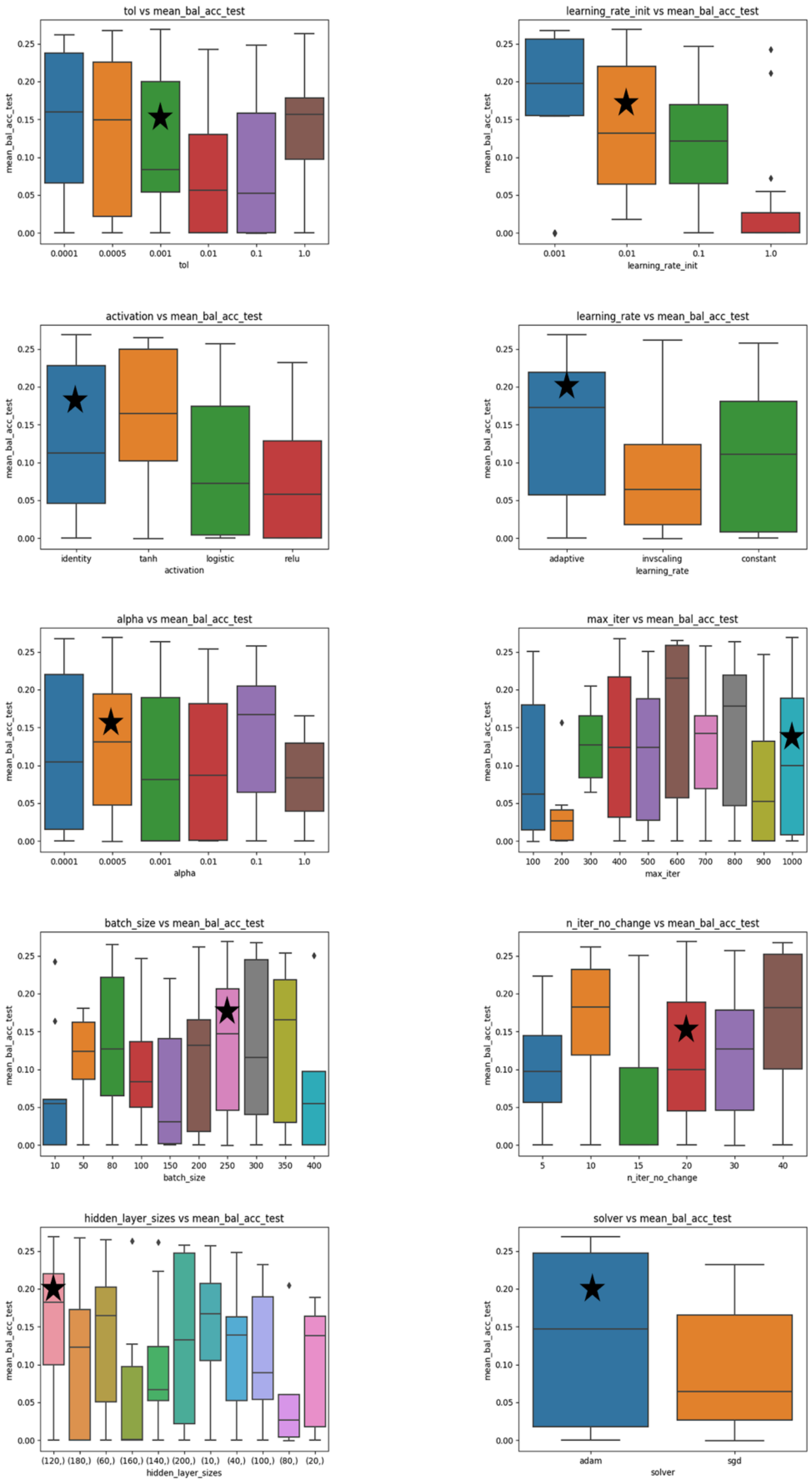

Supplementary Figure S4: Hyperparameter tuning parameter distribution. For each parameter, the star indicates the value chosen for the model.

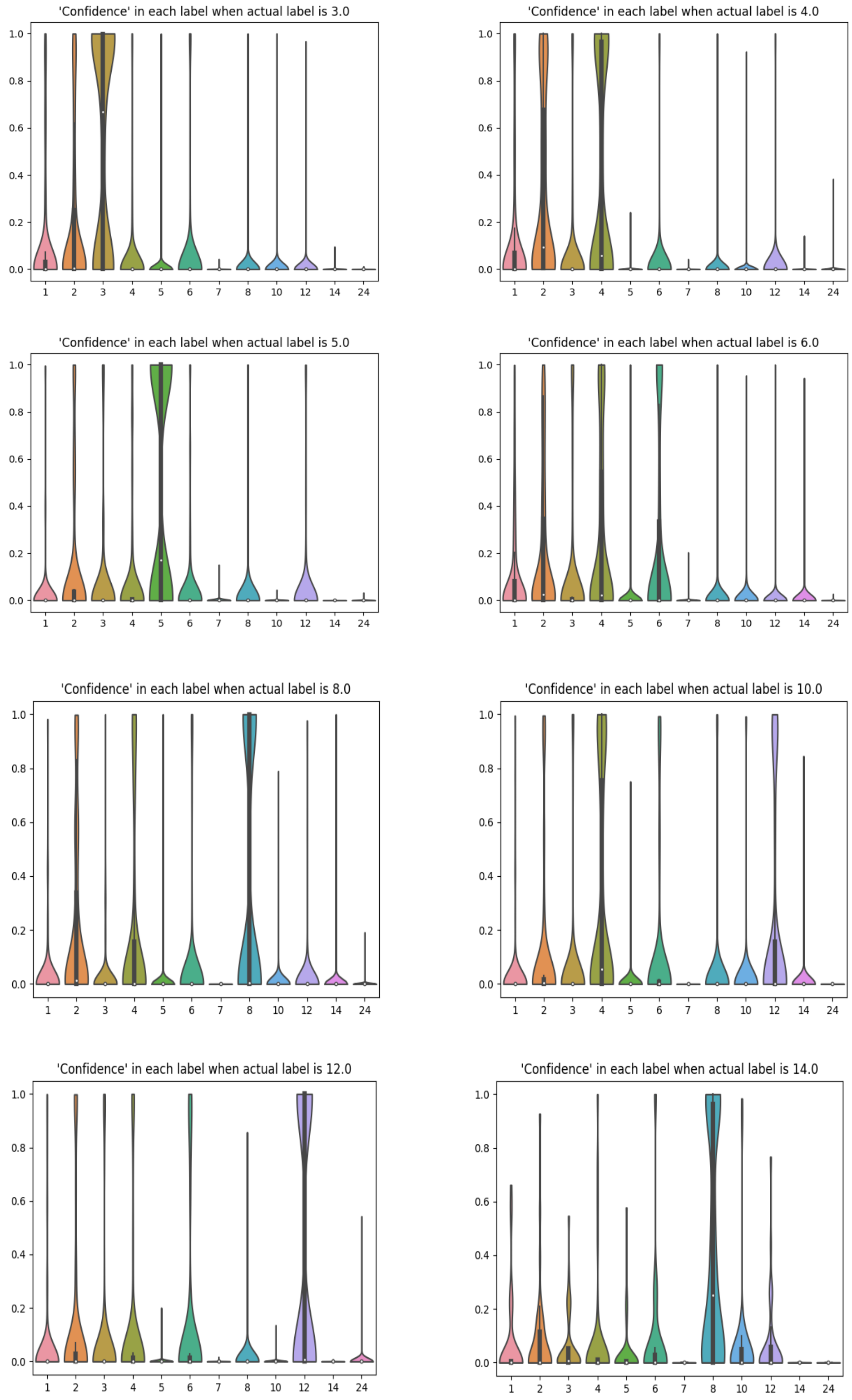

Supplementary Figure S5: Distribution of predicted probability (‘confidence’) for representative qs categories.

The plots show violin plots of the prediction probabilities (y-axis) for different qs labels (x-axis), for actual qs=3,4,5,6,8,10,12 and 14. Accompanies Figure 7.

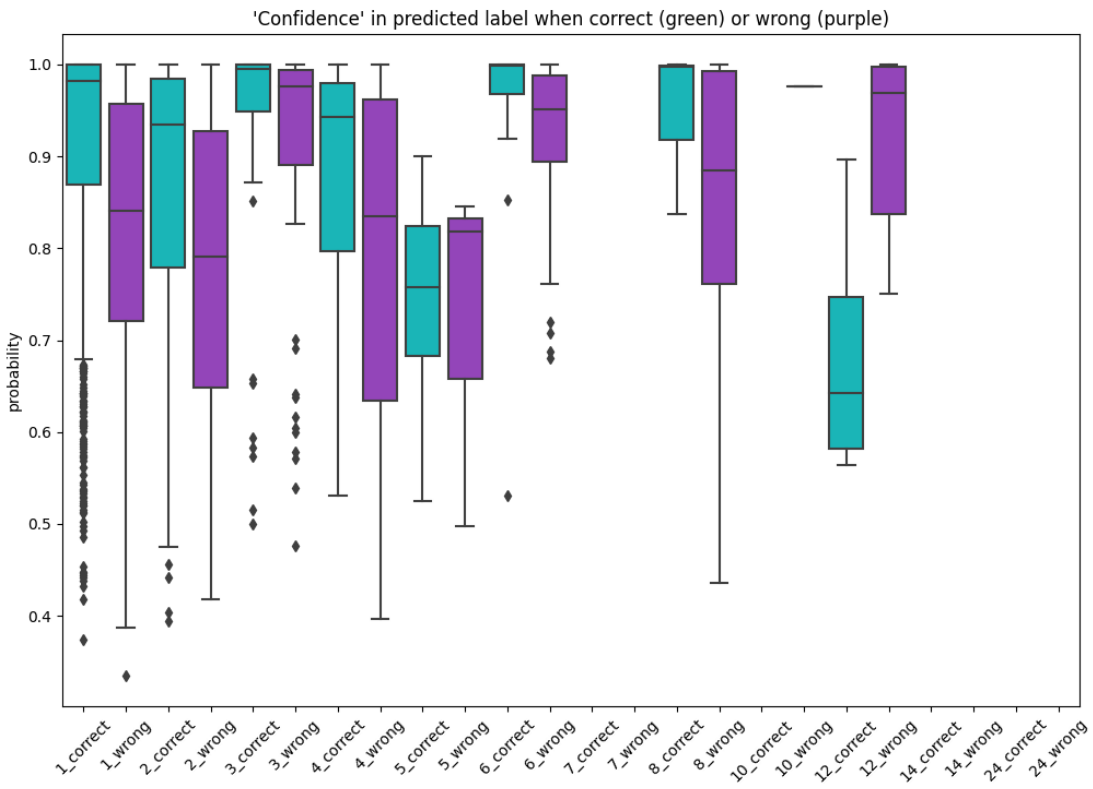

Supplementary Figure S6: QUEEN predictions show higher confidence for correct assignments also for the hold out set. Accompanies Figure 10.

For each qs (x-axis) box plots and dots show the distribution of the probabilities of correct (green) and incorrect (purple) predictions. Correct predictions are consistently predicted with higher probability than incorrect predictions, for most qs. This separation can be used to assess correct predictions, also in the absence of homologs.

Supplementary Tables

Supplementary Tables I and II are attached as tsv files.

Supplementary Table III - precision and recall per qs and overall				
qs	precision	recall	f1-score	support
1	0.66	0.71	0.69	4593
2	0.39	0.47	0.43	3384
3	0.55	0.54	0.55	1217
4	0.55	0.4	0.47	2743
5	0.72	0.48	0.58	108
6	0.27	0.23	0.25	816
7	1	0.05	0.09	21
8	0.48	0.38	0.43	307
10	0.04	0.03	0.04	96
12	0.26	0.4	0.31	171
14	0	0	0	37
24	0.97	0.59	0.74	125
macro average	0.49	0.36	0.38	13618
weighted average	0.53	0.52	0.52	13618

Supplementary Table IV(a) - Wilcoxon statistics and P-values of full homology annotation transfer		
qs	statistic	p-value
1	38.66	0
2	35.44	2.05E-275
3	14.40	2.46E-47
4	25.92	1.96E-148
5	5.02	2.61E-07
6	14.14	1.10E-45
7	1.64	0.051
8	10.10	2.69E-24
9	1.96	0.025
10	5.00	2.89E-07
11	nan	nan
12	5.82	2.95E-09
13	nan	nan
14	2.93	0.002
15	nan	nan
16	2.24	0.013
18	nan	nan
24	3.74	9.10E-05
60	nan	nan

Supplementary Table IV(b) - Wilcoxon statistics and P-values of no homology information annotation transfer		
qs	statistic	p-value
1	1.84	0.033
2	6.20	2.82E-10
3	2.90	0.002
4	3.38	3.56E-04
5	0.93	0.177
6	0.97	0.165
7	nan	nan
8	0.00	0.500
10	nan	nan
12	1.47	0.071
14	nan	nan
24	nan	nan

Supplementary Table V - Wilcoxon statistics and P-values of QUEENI		
qs	statistic	p-value
1	19.84	6.30E-88
2	8.83	5.40E-19
3	11.63	1.46E-31
4	12.21	1.35E-34
5	4.87	5.71E-07
6	4.84	6.53E-07
7	nan	nan
8	7.36	9.50E-14
10	2.59	0.005
12	8.85	4.33E-19
14	nan	nan
24	1.75	0.040
